# Associations between sugar-sweetened beverages before and during pregnancy and offspring overweight/obesity in Japanese women: the TMM BirThree Cohort Study

**DOI:** 10.1017/S1368980023000307

**Published:** 2023-06

**Authors:** Misato Aizawa, Keiko Murakami, Yudai Yonezawa, Ippei Takahashi, Tomomi Onuma, Aoi Noda, Fumihiko Ueno, Fumiko Matsuzaki, Mami Ishikuro, Taku Obara, Shinichi Kuriyama

**Affiliations:** 1 Graduate School of Medicine, Tohoku University, Aoba-Ku, Sendai, Miyagi, Japan; 2 Tohoku Medical Megabank Organization, Tohoku University, 2-1 Seiryo-Machi, Aoba-Ku, Sendai, Miyagi 980-8573, Japan; 3 Innovation Division, Kagome Co., Ltd., Nasushiobara-Shi, Tochigi, Japan; 4 Department of Pharmaceutical Sciences, Tohoku University Hospital, Aoba-Ku, Sendai, Miyagi, Japan; 5 International Research Institute of Disaster Science, Tohoku University, Aoba-Ku, Sendai, Miyagi, Japan

**Keywords:** Sugar-sweetened beverages, Offspring overweight/obesity, Pregnancy, Japan

## Abstract

**Objective:**

The association between high sugar-sweetened beverages (SSB) intake during pregnancy and offspring overweight/obesity has been reported only from Western countries. The objective of this study was to examine the association between SSB intake before and during pregnancy and offspring overweight/obesity among Japanese women.

**Design:**

Japanese prospective birth cohort study.

**Setting:**

We analysed mother–offspring pairs who participated in the Tohoku Medical Megabank Project Birth and Three-Generation Cohort Study from 2013 to 2017. SSB intake during pregnancy was evaluated using the FFQ and classified into three groups: none (0 g/d), medium (<195 g/d) and high (>195 g/d). Overweight or obesity at 1 year of age in offspring was defined as having a BMI *Z*-score greater than 2 sd, calculated based on the BMI reference data for Japanese children. Multiple logistic regression analyses were performed to examine the associations between SSB intake before and during pregnancy and offspring overweight/obesity, after adjusting for covariates.

**Participants:**

Japanese mother–offspring pairs (*n* 7114).

**Results:**

The overweight/obesity rate of the offspring was 8·8 %. Pregnant women with a high intake of SSB in early to mid-pregnancy had a higher risk of overweight/obesity in their offspring compared with those who did not; the OR was 1·52 (95 % CI (1·09, 2·12)).

**Conclusions:**

High SSB intake in early to mid-pregnancy was associated with an increased risk of offspring overweight/obesity at 1 year of age.

The incidence of childhood obesity has reached an alarming level worldwide^(1)^. In 2014, it was estimated that 41 million children under the age of 5 years were at risk of being overweight or obese^(1)^. In 2012, the prevalence of overweight among 3-year-olds in Japan was 5·1 %^(2)^. Childhood obesity is associated with an increased risk of poor academic performance^(3)^, depression, low self-esteem, bullying^(4)^; and chronic diseases such as adult obesity^(5)^, diabetes^(6,7)^, CVD, and cancer^(7)^. Furthermore, overweight/obesity at 1 year of age has been found to be associated with adult obesity^(8)^. Therefore, it is important to prevent obesity from an early age to avert lifestyle-related diseases in adults.

Adequate nutrition before and during early pregnancy has a positive impact on maternal and offspring health^(9)^. In contrast, undernutrition or overnutrition during pregnancy can lead to offspring overweight/obesity^(10)^. Therefore, pregnant women require proper nutrition and a healthy diet. High intake of artificially sweetened beverages (ASB) and sugar-sweetened beverages (SSB) in pregnant women has been shown to be associated with overweight/obesity in their children; studies conducted in Western countries showed that high ASB or SSB intake during pregnancy was associated with an increased risk of overweight/obesity after the first year of life^(11–16)^, and not with the birth weight of the child^(11,14)^. However, to our knowledge, no study has examined this association in Japan. Japanese adults have a lower soft drink consumption than adults from Western countries^(17)^. Therefore, it is possible that the SSB intake in Japanese pregnant women is lower than that in Western countries, and that its association with offspring overweight/obesity may differ from that observed in previous studies. Furthermore, it is still unclear at which stage of pregnancy SSB intake is associated with offspring overweight/obesity. Previous studies have reported associations of SSB consumption during early pregnancy^(13)^ and SSB consumption during mid-pregnancy with offspring overweight/obesity^(12,16)^.

Based on the above circumstances, this study aimed to examine the association between SSB intake and offspring overweight/obesity at 1 year of age in Japanese women while taking into account the timing of SSB intake.

## Methods

### Study design and population

We used data from the Tohoku Medical Megabank Project Birth and Three-Generation Cohort Study (TMM BirThree Cohort Study). Details of the TMM BirThree Cohort Study have been provided elsewhere^(18,19)^. Pregnant women and their families from Miyagi Prefecture were recruited for the TMM BirThree Cohort Study from July 2013 to March 2017. Approximately fifty obstetric clinics and hospitals participated in the recruitment process.

**Fig. 1:**
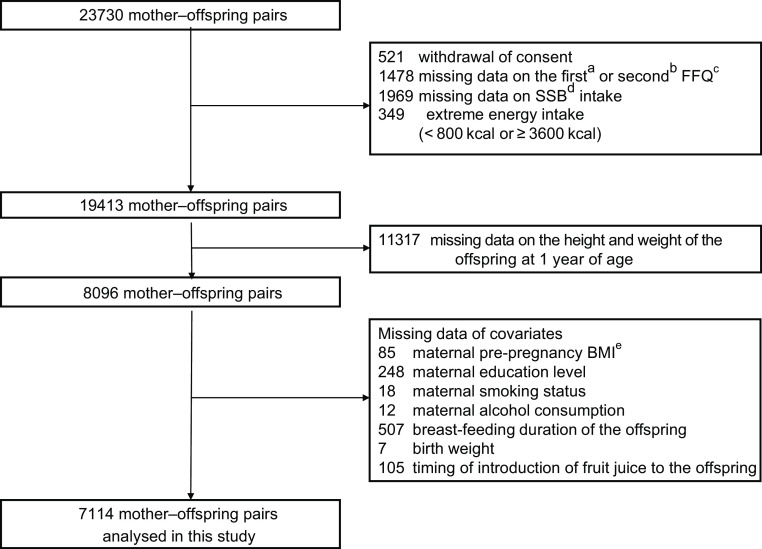
Flow chart of participant inclusion. ^a^The first FFQ was administered in early pregnancy to assess the frequency and quantity of foods and beverages consumed in the past year. ^b^The second FFQ was administered in mid-pregnancy to assess the frequency and quantity of foods and beverages consumed since the administration of the first FFQ. ^c^FFQ, food frequency questionnaire. ^d^SSB, sugar-sweetened beverages. ^e^BMI, body mass index.

Of the 23 730 mother–offspring pairs originally enrolled, we excluded 3990 mother–offspring pairs because of withdrawal of consent, missing data from the two FFQ administered, extreme energy intake (< 800 kcal or >3600 kcal) identified via the two FFQ^(20)^. Of the remaining 19 413 mother–offspring pairs, 11 317 were excluded because of missing data on the offspring’s height and weight at 1 year of age. Of the remaining 8096 mother–offspring pairs, 982 were excluded because of missing data on the maternal pre-pregnancy BMI, education level, smoking status and alcohol consumption; breast-feeding duration; offspring birth weight; and the timing of introduction of fruit juice to the offspring. The remaining 7114 mother–offspring pairs were included for analysis (Fig. 1). Differences in characteristics between the 16 616 pairs of mothers and offspring excluded from the analysis and the 7114 pairs included in the analysis are shown in Appendix A.

### Exposure variables

Intake of SSB and other foods was assessed using a 130-item self-administered semiquantitative FFQ^(21–23)^. Compared with the FFQ used in the Japan Public Health Centre-Based Prospective (JPHC) study, the response option ‘constitutionally unable to eat/drink’ was added to the FFQ used in this study^(21–23)^. The JPHC FFQ has been validated in the general Japanese population^(21–24)^, and the Spearman coefficient of validity for soft drinks (including cola and energy drinks) was 0·41 for women^(24)^. Data on SSB intake before and during pregnancy were obtained from the first and second FFQ, which were administered in early pregnancy (0–13 weeks of gestation) and mid-pregnancy (14–27 weeks of gestation), respectively. The mean response times for the first and second FFQ were 20·2 ± 7·2 and 28·3 ± 5·2 weeks of gestation, respectively. The first FFQ was administered in early pregnancy and assessed the frequency and amount of food items and beverages over the past year to evaluate food and beverage consumption from pre- to early pregnancy. The second FFQ assessed the frequency and quantity of foods and beverages consumed since the administration of the first FFQ. SSB was defined as the sum of soft drinks (e.g. cola), lactic acid bacteria beverages (e.g. Yakult), energy drinks (e.g. Lipovitan D) and canned coffee^(25)^. The frequency of intake of each beverage was evaluated using a questionnaire with the following ten items: ‘constitutionally unable to drink’, ‘never or < 1 cup a week’, ‘1–2 cups a week’, ‘3–4 cups a week’, ‘5–6 cups a week’, ‘1 cup a day’, ‘2–3 cups a day’, ‘4–6 cups a day’, ‘7–9 cups a day’ and ‘≥ 10 cups a day’. SSB intake was calculated by summing the intake of each beverage. SSB intake was adjusted for energy intake by summing the mean SSB intake and residuals^(26)^. For the categorical analysis, to examine the risk of being overweight and obese in children in the high SSB intake group (i.e. 90th percentile or higher) and SSB intake was divided into three categories: none (0 g/d), medium (< 195 g/d) and high (> 195 g/d) intake.

### Outcome variables

Height and weight data from the health check-up of the infants at 1 year of age were used; the BMI *Z*-score was calculated based on the BMI reference data for Japanese children^(27)^. Referring to the WHO cut-off value for overweight/obesity for children aged under 5 years, we defined offspring overweight/obesity as a BMI *Z*-score greater than 2 sd above at 1 year of age^(28)^.

### Potential confounders

Potential confounders were selected based on previous studies^(11–14)^. Maternal pre-pregnancy BMI (kg/m^2^) was calculated by dividing the pre-pregnancy weight (kg) by the square of the pre-pregnancy height (m^2^). It was divided into three categories: < 18·5 kg/m^2^, 18·5–24·9 kg/m^2^ and ≥ 25·0 kg/m^2^. Data regarding the maternal age at delivery (< 25 years, 25–29 years, 30–34 years or ≥ 35 years), history of gestational diabetes (ever or never) and parity (ever or never) were collected from the participants’ medical records. Data on smoking (never, quit before pregnancy, quit after pregnancy and current) were collected via a questionnaire administered in early pregnancy. Data on education level (high school or lower, junior college or vocational college, or university or higher) were collected 1 year after delivery. Total daily energy intake (continuous variable) was calculated using data from the Japanese Standard Tables of Food Composition (5^th^ edition, revised in 2005)^(29)^. Total consumption of cereals, potatoes, meat, sugar and alcoholic beverages was calculated from the FFQ and adjusted for energy (continuous variable). Offspring information, including birth weight (continuous variable), sex (male or female) and gestational age in weeks (continuous variable) were collected from medical records. The duration of breast-feeding (< 6 months or ≥ 6 months) was assessed via a questionnaire administered when the child was 1–6 months of age. Data on the timing of introduction of fruit juice to the offspring (age < 6 months, 7–8 months, 9–10 months, 11–12 months, not yet introduced) were collected 1 year after delivery.

### Statistical analysis

Maternal and offspring characteristics were compared according to maternal SSB intake using the ANOVA for continuous variables and the *χ* ^2^ test for categorical variables. The association between maternal SSB intake and the risk of offspring overweight/obesity at 1 year of age was assessed using multivariate logistic regression analysis, and OR or adjusted OR (aOR) and 95 % CI were calculated for each group compared with ‘None’. Three models were constructed to examine these associations. Model 1 was the crude model. Model 2 was adjusted for age at delivery and pre-pregnancy BMI. Model 3 was adjusted for the variables adjusted for in model 2 and education level; smoking status; parity; gestational diabetes; total energy intake; consumption of cereals, potatoes, meat, sugar, and alcoholic beverages; offspring sex; gestational age; birth weight; breast-feeding duration; and timing of introduction of fruit juice to the offspring. We calculated a trend *P*-value using the category of SSB intake as the continuous variable to estimate trends. As in previous studies^(11,12)^, the adjusted models were stratified to examine effect modification by maternal pre-pregnancy overweight/obesity and offspring sex. Maternal pre-pregnancy overweight/obesity was defined as a pre-pregnancy BMI ≥ 25·0 kg/m^2^. Interaction tests were conducted by entering the interaction terms in the logistic model. Statistical significance was set at *P* < 0·05. All statistical analyses were performed using SAS version 9.4 (SAS Institute Inc.).

## Results

Table 1 shows the characteristics of participants according to the SSB intake pre- to early pregnancy. The mean (sd) age at delivery was 32·4 (4·7) years, 10·4 % of the pregnant women were obese (BMI ≥ 25·0 kg/m^2^), and 8·8 % of the offspring were overweight/obesity. The mean (sd) SSB intake was 47·9 (121·1) g from pre- to early pregnancy and 36·1 (98·9) g from early to mid-pregnancy. Pregnant women with high SSB intake pre- to early pregnancy were younger, had higher pre-pregnancy BMI, had a lower education level, were more likely to have smoked during pregnancy, had shorter breast-feeding periods, and consumed fewer cereals, potatoes, fats, oils, and alcoholic beverages compared with pregnant women who did not consume SSB.

**Table 1:** Maternal and offspring characteristics according to maternal SSB intake: the TMM BirThree Cohort Study, 2013–2017, Japan

	SSB intake pre- to early pregnancy (g/d)						*P*-value†
	None*		Medium*		High*		
			Mean	sd	Mean	sd	
	0		51·5	48·3	262·7	217·6	
	*n* 992		*n* 5410		*n* 712		
Maternal characteristics	*n* or mean	% or sd	*n* or mean	% or sd	*n* or mean	% or sd	
Age at delivery (years)							
< 25	23	2·3	256	4·7	53	7·4	<0·001
25–29	162	16·3	1252	23·1	211	29·6	
30–34	412	41·5	2079	38·4	262	36·8	
≥ 35	395	39·8	1823	33·7	186	26·1	
Pre-pregnancy BMI‡ (kg/m^2^)							
<18·5	160	16·1	760	14·1	103	14·5	<0·001
18·5–24·9	752	75·8	4083	75·5	513	72·1	
≥ 25·0	80	8·1	567	10·5	96	13·5	
Educational level							
High school or lower	245	24·7	1556	28·8	307	43·1	<0·001
Junior college or vocational college	414	41·7	2163	40·0	253	35·5	
University or higher	333	33·6	1691	31·3	152	21·4	
Smoking status							
Never	685	69·1	3555	65·7	353	49·6	<0·001
Quit before pregnancy	221	22·3	1295	23·9	177	24·9	
Quit after pregnancy	77	7·8	499	9·2	142	19·9	
Current	9	0·9	61	1·1	40	5·6	
Parity ≥ 1	594	59·9	2752	50·9	325	45·7	<0·001
Gestational diabetes	23	2·3	61	2·1	19	2·7	0·55
Dietary intake§							
Energy (kcal/d)	2137	445	1541	453	1756	555	<0·001
Cereal (g/d)	426·9	133·1	426·9	103·8	396·1	116·2	<0·001
Potato (g/d)	25·7	23·3	24·8	24·0	23·2	19·6	<0·001
Meat (g/d)	73·7	54·2	74·9	33·7	73·5	41·7	<0·001
Oil and fat (g/d)	10·5	6·4	10·1	3·7	9·4	4·2	<0·001
Alcoholic beverage (g/d)	101·0	300·9	111·5	230·6	95·5	276·8	<0·001
Confectionary (g/d)	18·7	30·2	19·2	16·1	20·7	19·0	<0·001
Offspring characteristics							
Sex							
Male	506	51·0	2806	51·9	358	50·3	0·67
Female	486	49·0	2604	48·1	354	49·7	
Birth weight (g)	3040	397	3021	414	3012	436	0·34
Gestational age (weeks)	38·8	1·4	38·7	1·6	38·8	1·5	0·44
Breast-feeding duration ≥ 6 months	862	86·9	4436	82·0	533	74·9	<0·001
Timing of introduction of fruit juice to the offspring (age in months)							
< 6	327	33·0	1778	32·9	266	37·4	0·06
7–8	319	32·2	1763	32·6	233	32·7	
9–10	122	12·3	750	13·9	90	12·6	
11–12	119	12·0	545	10·1	75	10·5	
Not yet introduced	105	10·6	574	10·6	48	6·7	

SSB, sugar-sweetened beverages.

* None: 0 g/d, Medium: <195 g/d, High: ≥195 g/d.

† Obtained using the Student’s *t* test for continuous variables and the *χ* ^2^ test for categorical variables, comparing mother–offspring pairs who were analysed and mother–offspring pairs who were not.

‡ BMI *Z*-score was calculated based on the BMI reference data for Japanese children. Overweight/obesity was defined as a BMI *Z*-score greater than 2 sd above at 1 year of age.

§ All dietary intakes were energy-adjusted by the residual method. SSB included soft drinks, lactic acid bacteria beverages, energy drinks and canned coffee.

Table 2 shows the association between maternal SSB intake and offspring overweight/obesity at 1 year of age. Pregnant women who consumed high amounts of SSB pre- to early pregnancy had a higher risk of offspring overweight/obesity at 1 year of age compared with those who did not consume SSB (OR 1·48; 95 % CI (1·07, 2·06)) (Model 1). After adjustment for confounders, this association was no longer present; the multivariate-adjusted OR (95 % CI) were 1·37 (0·98, 1·90) (model 2) and 1·36 (0·96, 1·92) (model 3). Pregnant women who consumed high amounts of SSB early to mid-pregnancy also demonstrated a higher risk of offspring being overweight/obesity at 1 year of age compared with those who did not consume SSB (OR 1·68; 95 % CI (1·22, 2·30)) (model 1); this association was also present after adjusting for potential confounders: the multivariate-adjusted OR (95 % CI) were 1·56 (1·13, 2·15) (model 2) and 1·52 (1·09, 2·12) (model 3).

**Table 2:** Maternal SSB intake before and during pregnancy and offspring overweight/obesity* at 1 year of age: the TMM BirThree Cohort Study, 2013–2017, Japan

SSB intake	Offspring overweight/obesity*		Model 1		Model 2		Model 3	
	*N*/*n*	%	OR‡	95 % CI	OR‡	95 % CI	OR‡	95 % CI
Pre- to early pregnancy								
None†	992/79	8·0	1·00		1·00		1·00	
Medium†	5410/463	8·6	1·08	0·84, 1·39	1·03	0·81, 1·33	1·06	0·81, 1·40
High†	712/81	11·4	1·48	1·07, 2·06	1·37	0·98, 1·90	1·36	0·96, 1·92
*P*_for trend_ §			0·02		0·08		0·08	
Early to mid-pregnancy								
None†	1094/83	7·6	1·00		1·00		1·00	
Medium†	5309/454	8·6	1·14	0·89, 1·45	1·10	0·86, 1·40	1·11	0·85, 1·45
High†	711/86	12·1	1·68	1·22, 2·30	1·56	1·13, 2·15	1·52	1·09, 2·12
*P*_for trend_ §			0·002		0·01		0·01	

aOR: adjusted OR.

* BMI *Z*-score was calculated based on the BMI reference data for Japanese children. Overweight/obesity was defined as a BMI *Z*-score greater than 2 sd above at 1 year of age.

† None: 0 g/d, Medium: <195 g/d, High: ≥195 g/d.

‡ Multivariate logistic regression analysis adjusted for age at delivery, pre-pregnancy BMI, education; smoking status; parity; gestational diabetes; energy intake; intake of cereals, potatoes, meat, sugar, and alcoholic beverages; offspring sex; gestational age; birth weight; breast-feeding duration; and timing of introduction of fruit juice to the offspring.

§ *P*_for trends_ were calculated as trends across categories.

Table 3 shows the results of the stratified analysis conducted to examine whether the association between maternal SSB intake before and during pregnancy and offspring overweight/obesity differed by maternal pre-pregnancy overweight/obesity and offspring sex. In the stratified analysis by maternal pre-pregnancy BMI, the effects of pre- to early pregnancy maternal SSB consumption on offspring overweight/obesity were similar among normal weight (aOR 1·30; 95 % CI (0·89, 1·90)) and overweight/obese mothers (aOR 1·82; 95 % CI (0·73, 4·51)) (*P* = 0·46 for the interaction). The effect of maternal SSB consumption from early to mid-pregnancy was also similar for normal weight (aOR 1·31; 95 % CI (0·98, 2·25)) and overweight/obese (aOR 1·35; 95 % CI (0·54, 43·40)) mothers (*P* = 0·85 for the interaction). In stratified analyses by offspring sex, the effects of maternal SSB consumption pre- to early pregnancy on offspring overweight/obesity were similar for males (aOR 1·28; 95 % CI (0·80, 2·06)) and females (aOR 1·47; 95 % CI (0·88, 2·47)) (*P* = 0·96 for the interaction). The effect of maternal SSB consumption from early to mid-pregnancy on offspring overweight/obesity was observed only for female infants, males (aOR 1·13; 95 % CI (0·71, 1·81)) and females (aOR 2·17; 95 % CI (1·34, 3·54)) (*P* = 0·19 for the interaction).

**Table 3:** Maternal SSB intake before and during pregnancy and offspring overweight/obesity* stratified by pre-pregnancy BMI and offspring sex: the TMM BirThree Cohort Study, 2013–2017, Japan

SSB intake	Pre- to early pregnancy				Early to mid-pregnancy			
	*N*/*n*	%	aOR‡	95 % CI	*N*/*n*	%	aOR‡	95 % CI
Pre-pregnancy BMI								
<25·0								
None†	912/70	7·7	1·00		1007/74	7·4	1·00	
Medium†	4843/391	8·1	1·09	0·82, 1·46	4758/378	7·9	1·07	0·81, 1·43
High†	616/62	10·1	1·30	0·89, 1·90	606/71	11·7	1·31	0·98, 2·25
≥25·0								
None†	80/9	11·3	1·00		87/9	10·3	1·00	
Medium†	567/72	12·7	0·96	0·43, 2·14	551/76	13·8	1·32	0·60, 2·91
High†	96/19	19·8	1·82	0·73, 4·51	105/15	14·3	1·35	0·54, 3·40
*P*_for interaction_ §			0·46				0·85	
Offspring sex								
Male								
None†	506/36	7·1	1·00		557/36	6·5	1·00	
Medium†	2806/224	8·0	0·99	0·68, 1·44	2753/214	7·8	1·03	0·72, 1·49
High†	358/36	10·1	1·28	0·80, 2·06	360/46	12·8	1·13	0·71, 1·81
Female								
None†	486/43	8·9	1·00		537/47	8·8	1·00	
Medium†	2604/239	9·2	1·13	0·76, 1·69	2556/240	9·4	1·26	0·84, 1·89
High†	354/45	12·7	1·47	0·88, 2·47	351/40	11·4	2·17	1·34, 3·54
*P*_for interaction_ §			0·96				0·19	

aOR, adjusted OR.

* BMI *Z*-score was calculated based on the BMI reference data for Japanese children. Overweight/obesity was defined as a BMI *Z*-score greater than 2 sd above at 1 year of age.

† None: 0 g/d, Medium: <195 g/d, High: ≥195 g/d.

‡ Multivariate logistic regression analysis adjusted for age at delivery, pre-pregnancy BMI, education; smoking status; parity; gestational diabetes; energy intake; intake of cereals, potatoes, meat, sugar, and alcoholic beverages; offspring sex; gestational age; birth weight; breast-feeding duration; and timing of introduction of fruit juice to the offspring.

§ *P* values were determined using the interaction test.

## Discussion

This study examined the association between SSB intake before and during pregnancy and offspring overweight/obesity in Japanese women. SSB intake in early to mid-pregnancy was associated with an increased risk of offspring overweight/obesity at 1 year of age.

In studies conducted in Canada^(11)^ and Denmark^(14)^, SSB intake during pregnancy was not associated with an increased risk of overweight/obesity at ages 1 and 7 years in offspring. In contrast, studies conducted in the USA^(12,16)^ and the Netherlands^(13)^ showed that high SSB intake during pregnancy was associated with an increased risk of offspring overweight/obesity at ages 6 and 7 years and increased energy intake. Our result is consistent with the associations observed in the previous studies of the latter.

A previous study comparing the average SSB intake among adults from thirteen countries found that Japanese adults consumed fewer SSB than those from Western countries and similar levels in Asian countries (110 ml/d in Japan, 370 ml/d in the UK and 100 ml/d in China)^(17)^. Regarding SSB intake among pregnant women, 23·4 % of pregnant women in Canada and 19·8 % of pregnant women in the USA consumed at least one cup of SSB per d^(11,12)^. The present study showed that the average SSB intake among Japanese pregnant women, calculated from the FFQ, was 47·9 g/d during pre- to early pregnancy and 36·1 g/d during early to mid-pregnancy, suggesting that the SSB intake among pregnant women is lower in Japan than that in Western countries. However, we demonstrated that even in Japan, higher SSB intake during pregnancy was associated with an increased risk of overweight/obesity in the offspring.

The mechanism through which SSB intake in early to mid-pregnancy is associated with offspring overweight/obesity may be as follows: prenatal nutrition is thought to play an important role because predisposition to metabolic diseases may be programmed during the fetal period^(30)^. Among foods, SSB are among the most sugar-rich foods^(31)^. Studies in mice have shown that a high intake of sugar-rich SSB before and during pregnancy alters epigenetics and is associated with hypertension, obesity, and insulin resistance in the offspring^(32,33)^. Although the mechanism by which sugar intake during pregnancy in humans affects the health of the offspring is not clear^(33)^, previous studies^(12,13,16)^ and the results of this study suggest that high sugar content in SSB may have affected the offspring’s overweight/obesity. Controlling for confounding factors did not alter the observed association between maternal SSB intake and offspring overweight/obesity at 1 year of age, suggesting that *in utero* SSB exposure has an independent effect. In addition, mothers’ unhealthy parenting style may have affected their offspring’s weight (i.e. being overweight/obese), since SSB is often considered an indicator of a mother’s unhealthy lifestyle^(34)^; however, we found an association between high SSB intake during pregnancy and offspring being overweight/obese even after adjusting for unhealthy behaviours such as smoking and alcohol intake.

The present study found that SSB intake in early to mid-pregnancy was associated with offspring being overweight/obese, while intake in pre- to early pregnancy was not. To our knowledge, there is only one previous study comparing the timing of SSB consumption during pregnancy and offspring weight and found that SSB consumption in mid-pregnancy was associated with increased BMI in 7-year-old offspring, but not SSB consumption in early pregnancy^(12)^. The first FFQ used in the present study was conducted in early pregnancy and assessed dietary intake before and through early pregnancy. Thus, the findings of our current study, similar to those of Gillman *et al.* ^(12)^, suggest that SSB intake from early to mid-pregnancy may have a greater impact on offspring weight than from pre- to early pregnancy.

As reported in previous studies^(12,13)^, the association between SSB intake before and during pregnancy and offspring overweight/obesity was not significantly affected by maternal preconception BMI or offspring sex. In a Dutch study^(13)^, higher maternal SSB intake was associated with higher weight in female offspring, which was mainly due to the consumption of 100 % fruit juice. In a study with mice^(35)^, when mothers consumed high fructose, the main ingredient in SSB, female offspring were more prone to excess fat mass accumulation and excess weight gain, while male offspring were more prone to insulin resistance and hyperleptinemia and hypoadiponectinemia. The findings of this study suggest that high SSB intake during pregnancy may affect only the weight gain of the female offspring. The *P* for the interaction by offspring sex was not significant, although it may have been underpowered to test this interaction conclusively.

### Implications

The findings of this study suggest that women should reduce their SSB intake during early to mid-pregnancy to prevent overweight/obesity in their offspring, even in Japan, where SSB intake is lower than that in Western countries. Maternal nutrition and diet before and during pregnancy affect the health and growth of the offspring^(10)^. Pregnancy provides a good opportunity for women to review their unhealthy eating habits, especially as it is easy to change their eating habits during pregnancy^(36)^, and nutritional advice is more likely to be followed if it is given in terms of specific foods rather than nutrients^(37)^. Considering the findings of this study, interventions focusing on discouraging pregnant women from consuming SSB may be effective for the prevention of overweight/obesity in children.

### Limitations

There are several limitations to this study. First, the FFQ used in this study was modified version of the FFQ used in the JPHC study. The FFQ used in the JPHC study has been validated in Japanese population^(21–23)^. The FFQ in this study only added the option ‘constitutionally unable to eat/drink’ to the FFQ in the JPHC study; the option was treated as equivalent to ‘never eat/drink at all’ or ‘less than once a month’, when the intake of each food item was calculated. Therefore, it would have little impact on the interpretation of the results. Second, the intake of each beverage was assessed by a self-administered questionnaire, which could lead to inaccurate responses due to participant error in judgement. Third, the first FFQ was administered during early pregnancy and assessed the frequency and quantity of foods and beverages in the past year to assess dietary intake from pre- to early pregnancy. Although participants may have changed their dietary habits as a result of pregnancy, we were unable to assess the impact of dietary changes on the results of this study. Fourth, many mother–offspring pairs were excluded from the analysis, which may introduce selection bias. Compared with mothers included in the analysis, excluded mothers were younger and had higher pre-pregnancy BMI, lower levels of education, and higher rates of smoking during pregnancy. Compared with the children included in the analysis, children excluded from the analysis had shorter gestational weeks, lower birth weights, shorter breast-feeding periods and higher rates of overweight/obesity. Thus, the association between maternal SSB intake and offspring overweight/obesity in the present study may have been overestimated. Fifth, the TMM BirThree Cohort Study was conducted in one of the forty-seven prefectures in Japan, which limits the generalisability of the findings.

## Conclusion

High intake of SSB in early to mid-pregnancy among Japanese pregnant women was associated with an increased risk of offspring overweight/obesity at 1 year of age. Therefore, reducing SSB intake during early to mid-pregnancy may help reduce childhood obesity in Japan.

## Supplementary material

For supplementary material accompanying this paper visit https://doi.org/10.1017/S1368980023000307. click here to view supplementary material
