# Supplementary material for: Associations between sugar-sweetened beverages before and during pregnancy and offspring overweight/obesity in Japanese women: the TMM BirThree Cohort Study
Source: Public Health Nutr. 2023 Feb 9;26(6):1222–9. doi: 10.1017/S1368980023000307 (PMC10346008; doi:10.1017/S1368980023000307)
Supplement: Supplementary file 1 [file S1368980023000307sup001.docx]

| **Appendix A. Characteristics differences between 16616 mother–offspring pairs who were analyzed** | | | | | |
| --- | --- | --- | --- | --- | --- |
| **and 7114 mother–offspring pairs who were excluded from the analysis** | | | |  |  |
| **:The TMM BirThree Cohort Study, 2013-2017, Japan** | | |  |  |  |
|  | **Mother–offspring pairs  who were analyzed (n=7114)** | | **Mother–offspring pairs  who were not analyzed (n=16616)** | | **p-value^a^** |
|  | **Mean ±SD or n (%)** | | | |  |
| **Maternal characteristics** |  | |  | |  |
| Age at delivery (years) | 32.4 | ±4.7 | 31.4 | ±5.2 | <0.001 |
| < 25 | 332 | (4.7) | 1557 | (9.3) |  |
| 25-29 | 1625 | (22.8) | 4467 | (26.9) |  |
| 30-34 | 2753 | (38.7) | 5927 | (35.7) |  |
| ≥ 35 | 2404 | (33.8) | 4665 | (28.1) |  |
| Pre-pregnancy BMI^b^ (kg/m^2^) | 21.3 | ±3.3 | 21.5 | ±3.5 | <0.001 |
| <18.5 | 1023 | (14.4) | 2396 | (14.4) |  |
| 18.5-24.9 | 5348 | (75.2) | 12062 | (72.6) |  |
| ≥ 25.0 | 743 | (10.4) | 2158 | (13.0) |  |
| Educational level |  |  |  |  |  |
| High school or lower | 2108 | (29.6) | 5955 | (35.8) | <0.001 |
| Junior college or vocational college | 2830 | (39.8) | 6213 | (37.3) |  |
| University or higher | 2176 | (30.6) | 4408 | (26.5) |  |
| Smoking status |  |  |  |  |  |
| Never | 4593 | (64.6) | 9550 | (57.4) | <0.001 |
| Quit before pregnancy | 1693 | (23.8) | 3838 | (23.1) |  |
| Quit after pregnancy | 718 | (10.1) | 2678 | (16.1) |  |
| Current | 110 | (1.6) | 550 | (3.3) |  |
| Parity ≥ 1 | 3671 | (51.6) | 8607 | (51.8) | 0.92 |
| Gestational diabetes | 154 | (2.2) | 432 | (2.6) | 0.12 |
| Total energy intake (kcal/day) | 1646 | ±507 | 1576 | ±618.1 | 0.25 |
| Cereals consumption (g/day) | 421.6 | ±110.2 | 425.1 | ±130.1 | 0.59 |
| Potato consumption (g/day) | 24.4 | ±17.9 | 24.8 | ±20.8 | <0.001 |
| Meat consumption (g/day) | 74.6 | ±38.0 | 76.0 | ±54.0 | 0.002 |
| Oil and fat consumption (g/day) | 9.9 | ±4.2 | 10.3 | ±5.3 | 0.80 |
| Alcoholic beverage consumption (g/day) | 108.5 | ±246.5 | 111.6 | ±230.2 | 0.19 |
| Confectionary consumption (g/day) | 18.8 | ±19.0 | 19.2 | ±20.3 | 0.003 |
| **Offsprring characteristics** |  |  |  |  |  |
| Sex |  |  |  |  |  |
| Male | 3670 | (51.6) | 8640 | (52.0) | 0.39 |
| Female | 3444 | (48.4) | 7976 | (48.0) |  |
| Overweight/obesity ^c^ | 623 | (8.8) | 1362 | (8.2) | <0.001 |
| Birthweight (g) | 3023 | ±414 | 3008 | ±480.8 | <0.001 |
| Gestational age (weeks) | 38.8 | ±1.6 | 38.6 | ±2.4 | <0.001 |
| Breastfeeding duration ≥ 6 months | 5831 | (82.0) | 12960 | (78.0) | <0.001 |
| Timing of introduction of fruit juice to the offspring (age in months) |  |  |  |  |  |
| < 6 | 2371 | (33.3) | 5587 | (33.6) | 0.86 |
| 7-8 | 2315 | (32.5) | 5352 | (32.2) |  |
| 9-10 | 962 | (13.5) | 2398 | (14.4) |  |
| 11-12 | 739 | (10.4) | 1324 | (8.0) |  |
| Not yet introduced | 727 | (10.2) | 1955 | (11.8) |  |
| BMI, body mass index |  |  |  |  |  |
| ^a^Obtained using the Student's *t* test for continuous variables and the chi-squared test for categorical | | | | | |
| variables, comparing mother-offspring pairs who were analyzed and mother-offspring pairs who were not. | | | | | |
| ^b^BMI was calculated by diving the pre-pregnancy weight (kg) by the square of the pre-pregnancy height (m^2^). | | | | | |
| ^c^BMI z-score was calculated based on the BMI reference data for Japanese children. Overweight/obesity was defined as a BMI z-score greater than 2 standard deviations above at 1 year of age. | | | | | |
